# Weight loss is associated with improved quality of life among rural women completers of a web-based lifestyle intervention

**DOI:** 10.1371/journal.pone.0225446

**Published:** 2019-11-19

**Authors:** Patricia A. Hageman, Joseph E. Mroz, Michael A. Yoerger, Carol H. Pullen

**Affiliations:** 1 Division of Physical Therapy Education, College of Allied Health Professions, University of Nebraska Medical Center, Omaha, Nebraska, United States of America; 2 College of Nursing-Omaha Division, University of Nebraska Medical Center, Omaha, Nebraska, United States of America; Vanderbilt University, UNITED STATES

## Abstract

**Introduction:**

The evidence for whether weight loss following longer-term lifestyle interventions results in improved health-related quality of life (HRQoL) is inconclusive. This study examines whether women who lose weight after completing an 18-month web-based lifestyle modification intervention would report a corresponding improvement in HRQoL as measured using the Patient-Reported Outcomes Measurement Information System 29-item profile (PROMIS-29 v1.0).

**Methods:**

Data from 216 rural women, ages 40 to 69, with baseline and 18-month PROMIS-29 data were analyzed in this secondary analysis of the Women Weigh-in for Wellness clinical trial. This trial promoted lifestyle modification for initial weight loss (baseline to 6 months) and guided weight loss (6 months to 18 months) using a web-delivery format.

**Results:**

After adjusting for age, number of comorbidities, change in physical activity from baseline, intervention group, and baseline PROMIS-29 scores, change in weight was associated with improved health-related quality of life (HRQoL) in the domains of depression, physical function, pain interference, fatigue, and satisfaction with social role. Logistic regressions, adjusting for the same factors, indicated women with ≥ 10% weight loss were more likely to report lower depression, higher physical function and less pain interference, compared to women who gained weight or lost < 5%.

**Conclusions:**

Among our rural women, a loss in weight from baseline appeared to be associated with overall improvement in multiple PROMIS-29 v 1.0 domains, noting the likelihood of achieving improvement was significantly higher among women who attained ≥ 10% weight loss. These findings may positively influence a woman’s adherence to lifestyle modification weight loss and weight maintenance program.

**Trial registration:**

ClinicalTrials.gov NCT01307644.

## Introduction

Obesity is a major public health challenge, considered as one of the leading preventable causes of chronic disease and death [1–2]. The prevalence of obesity is high across the United States, and individuals residing in rural compared with urban areas may be at higher risk of obesity-related behaviors [3–4]. Cross sectional studies provide evidence that obesity is associated with lower health-related quality of life (HRQoL) [5–6], especially among women [7– 8]. Obesity-related behaviors, such as poor dietary quality and a sedentary lifestyle, are associated with reduced physical function by both HRQoL self-report and objective measures [5,9]. Of major concern, a reduction in physical function may lead to higher prevalence of late-life disability, particularly among women compared with men [10]. HRQoL is considered a useful outcome for assessing an individual’s perceptions of the impact of disability on his/her physical, psychological, and social functioning [6].

While there is consistency among cross-sectional studies of the relationship between body mass index and HRQoL, the evidence is inconclusive as to whether weight loss achieved through longer-term lifestyle modification may result in improved HRQoL, as some trials observe improvements whereas others do not [11]. Weight loss following lifestyle interventions is linked with improvements in physical HRQoL, but it is associated with minimal or no improvements in social function or mental function HRQoL such as depression [11–14]. Greater weight loss is associated with increased or improved HRQoL, with the majority of improvements in HRQoL domains following a period of acute weight loss, typically after 6 to 9 months [12, 15].

Research about the potential influence of weight loss interventions focused on lifestyle modification on change in HRQoL among the general population is inconsistent and/or lacking [6, 16]. Inconsistences in these research findings are attributed to the variation between studies in the quality of reporting, the populations of study, the types of weight loss interventions, and the HRQoL assessment measures, with most available studies being of short duration [6,11,16]. Attaining a weight loss change of ≥ 5 and/or ≥ 10% of initial bodyweight is correlated with a reduction in cardiovascular disease risk and other comorbidities; however, whether this level of weight loss would increase the likelihood of improvements in HRQoL remains unclear due to limited studies and/or varying study findings [14,16–18].

This study adds to the literature as it measures the association between weight change and HRQoL as measured by the Patient-Reported Outcomes Measurement Information System (PROMIS-29 v1.0) among completers of an 18-month lifestyle modification intervention for weight loss and weight maintenance delivered by web. The PROMIS-29 is a relatively new instrument intended by the National Institutes of Health for use in as a short and generic, yet precise, assessment of health outcomes of individuals within the general population and those with chronic disorders and diseases [19–21]. The study uses data collected from an understudied sample of rural women considered a designated priority population for cardiovascular disease prevention by the US Department of Health and Human Services [22].

Specifically, this secondary analysis of clinical trial data examines whether women who lose weight after completing an 18-month web-based lifestyle modification intervention would report a corresponding improvement in their HRQoL as measured using the PROMIS-29 v1.0. The study also examines whether women with weight losses of 5% to 9.9% or ≥ 10% from baseline to 18-months would have a higher likelihood of improved HRQoL as compared to women who gained weight or had < 5% weight loss. Based on available literature, we hypothesized greater weight loss would be associated with improved HRQoL in the physical domain [13,14,16].

## Methods

Details about the methods, flow chart, and main effects of the Women Weigh-in for Wellness community-based randomized controlled trial, from which data were used for this secondary analysis, are described elsewhere [23,24]. In brief, the purpose of the parent trial was to compare the effectiveness of a lifestyle modification approach using web-delivery in midlife and older rural women randomized to a web-based only group or a web-based group supplemented with either a peer-led discussion board or professional email counseling. All intervention groups received access to the main website content, and the intervention consisted of 3 phases focusing on weight loss (phase 1), guided weight maintenance (phase 2), and self-directed weight maintenance and follow up (phase 3).

Analyses of clinical trial data revealed no intervention group differences in the primary trial outcome of weight [24], so data from women across all intervention groups were combined for the present analyses. For this secondary analysis, we examined women’s change in weight associated with change in HRQoL using data from phase 1 and phase 2 of the intervention, as phase 3 of the trial targeted self-directed weight maintenance and the use of the intervention website was optional. Our analysis was limited to 216 women who had complete data on age, health history, physical activity, weight, and PROMIS-29 profile data at both baseline and 18 months.

Recruitment occurred through mailings, flyers, and contacts as recommended by a community advisory board. Women were eligible to enroll if they were age 40–69 and had a body mass index of 28 to 45 kg/m^2^ as verified by objective measurement taken by trained research nurses. Women were eligible if they had access to and comfort with email and the internet, and were willing to drive up to 70 miles each way to a centrally located office for assessments. Women were excluded if they had a 10% weight loss within the past 6 months, were on medications that affected weight, had diabetes type 1 or type 2 requiring insulin, were enrolled in another weight loss program, or had physical or medical conditions that would prevent them from participating in the lifestyle modification plan.

Of 301 women enrolled in the parent trial, 230 completed 18 months, and 216 women had complete data needed for the analyses. Trained research nurses conducted all the assessments at baseline and 18 months at the centrally located office.

### Ethics statement

The study was approved by the Institutional Review Board of the University of Nebraska Medical Center and was conducted according to the principles expressed in the Declaration of Helsinki. All women provided written informed consent in accordance with the University of Nebraska Institutional Review Board (approval number: 23710-FB) prior to enrollment in the study.

### Intervention

Regardless of intervention group assignment, all women received access to the project’s website. The intervention website included an evidence-based lifestyle modification plan for healthy eating and activity, theory-based messaging including goal setting, and self-tracking of weight, food intake, and activity [24]. The healthy eating and activity plans were based upon the 2010 Dietary Guidelines for Americans [25], Healthy People 2010 recommendations [26], and the 2008 Physical Activity Guidelines for Americans [27]. Website messaging was developed based on constructs of Pender’s Health Promotion model [28], concentrating on benefits and overcoming barriers to, building self-efficacy and interpersonal support for eating and activity behavior change, and goal-setting [29].

During intervention phase 1 (baseline to 6 months), the focus was on guided weight loss to attain ≥ 5% initial bodyweight loss, with encouragement to lose > 10% initial bodyweight loss, as these targets are consistent with achieving important health benefits [17]. Women received 26 weeks of new messaging content, and they were encouraged to log weight, Kcal intake, and pedometer steps taken daily and goal setting weekly.

During phase 2 (6 months to 18 months), the intervention emphasis was on continued weight loss and guided weight-loss maintenance of ≥ 5% initial bodyweight. Messaging content concentrated on contemporary stories about weight loss found in social media, called “hot topics,” that were based on evidence. Women received a new “hot topic” message biweekly during 6 months to 12 months, and monthly during 12 months to 18 months. Women were asked to log weight weekly, and were encouraged to continue logging eating, activity and goals as in phase 1, or at a minimum of bi-weekly.

### Assessments

Weight (kg) and body mass index (kg/m^2^) were assessed using a Tanita scale (TBF-215, Tanita Corporation of America, Inc, Arlington Heights, Il, USA) following the manufacturer’s protocol.

Women were asked to complete surveys regarding their general demographic information and health histories. The health history questionnaire asked women whether they had ever been told by a physician that they had been diagnosed with medical conditions in the categories of arthritis, cancer, cardiovascular disease (including hypertension), diabetes, general health (includes depression and anxiety), organ disease, neurological, other muscular conditions, respiratory, and/or thyroid disease. Each woman’s number of affirmative responses of these categories were summed to yield a total score of number of comorbidities.

Physical activity was assessed using a standardized procedure using the Actigraph accelerometer (Model GT3X; ActiGraph, Pensacola, FL), a device which has high reliability and validity [30]. Women were asked to wear the device 24 hours a day, except during activities such as showering, over seven days, both at baseline and 18 months. It was placed on their dominant hip, and was secured to an elastic waist band. The device permitted an estimation of average daily minutes in moderate or greater intensity activity, using established cut-points [31].

Health-related quality of life was assessed using the Patient-Reported Outcomes Measurement System-29 (PROMIS-29) profile version 1.0 survey which was found to have reliability and validity in both the general and clinical U.S. populations [20,21]. The survey consists of four questions for each of seven distinct domains (anxiety, depression, fatigue, pain, physical function, sleep disturbance, and satisfaction with social role). With the exception of the physical function domain that focused on the present, all questions are based on a 7-day recall, and use a 5-point Likert-type response. The instrument is scored using T-scores, a standardized metric that has a mean of 50 following the standardized reporting method [20]. A higher PROMIS T-score on any given domain represents more of the concept being measured. Reliability estimates for internal consistency of our use of the PROMIS-29 ranged from α = .76 for physical function to α = .94 for pain interference (average reliability across domains was α = .87).

### Data analysis

All statistical analyses were completed using SPSS v 25. Descriptive statistics for demographics and other women’s data were calculated. Prior to conducting any analyses, we evaluated the normality of the data. Normality was assessed by examining frequency tables, the absolute value of skewness and kurtosis, histograms with normal curve overlays, and normal p-p plots for each assessment, using best practice recommendations for evaluating normality with samples of a similar size to the one used in this study [32,33]. Methods of statistically testing a distribution for normality, such as calculating a *z* score by dividing the skewness and kurtosis values by their standard errors or conducting the Kolmogorov-Smirnov or Shapiro-Wilk tests of normality, are highly sensitive to even very small deviations from normality and are not recommended for use with samples exceeding or equal to 300 [32,33]. None of the skewness or kurtosis values approached the cut-off of 2.00 proposed by Kim [32]. Based on all evaluations of the data, all scales were determined to have an adequate distribution for conducting parametric tests.

We used *t*-tests to compare the baseline PROMIS-29 scores of women who completed the study at 18 months and women who dropped out. Linear regression analyses were used to examine the effect of weight change on women’s change in PROMIS-29 domains from baseline to 18 months. These linear regression models controlled for variables that are clinically relevant to HRQoL and weight, including age, number of comorbidities, intervention group, physical activity, and baseline scores on the PROMIS-29 [34–36]. Unstandardized regression weights (*b*) with 95% confidence intervals (CIs) are presented throughout the results and associated tables. We estimated one regression model for each of the seven PROMIS-29 domains

In addition, we also conducted a series of logistic regression analyses designed to provide additional insight into the clinical relevance of the association between weight loss and change in quality of life. Women completers were classified into three categories of weight loss at 18 months, similar to Pearl and associates [18]: < 5% of initial bodyweight loss, 5% to 9.9% initial bodyweight loss, or ≥ 10% initial bodyweight loss. Mean change scores for weight and PROMIS-29 were calculated from baseline to 18 months. Logistic regression models were used to compare the three categories of weight loss based on those who did or did not achieve a minimal clinically important difference (MCID) of an intra-subject change of at least 6 or more points in the desired direction for each of the seven PROMIS-29 domains. This MCID for PROMIS-29 domains is consistent with the threshold used in another study of similar participant characteristics [37]. This is a conservative method of statistical analysis as our results are not biased by small, random fluctuations in HRQoL domain scores that might occur when treating HRQoL change as continuous variables. The referent category was < 5% of initial bodyweight loss from baseline that included women with weight gain.

The same control variables used in the linear regressions were adjusted for in the logistic analyses: age, number of comorbidities, intervention group, physical activity, and baseline scores on the PROMIS-29. As the parent trial was powered based on an accepted strategy of estimating a priori power only for the analysis of primary outcome of weight, we acknowledge the analyses of the PROMIS-29 measure as tertiary outcome in this study is likely to have less power, and is conducted for is descriptive value.

## Results

At baseline, the mean (SD) age was 54.7 (6.78) for the 216 women included in this analysis. These women had a baseline mean (SD) body mass index of 34.5 (4.23) k/m^2^. Baseline data of these 216 women revealed they were primarily Caucasian (98.1%; n = 212), employed either full- or part-time (84.3%; n = 182), had completed some college or higher (82.9%; n = 179), and reported a household income ≥ $40,000 (80.2%; n = 163). The majority of women were healthy, with 51 (23.6%) reporting no comorbidities, 80 (37.0%) having one comorbidity, and 85 (39.4%) reporting two or more comorbidities. Self-reported physician diagnosis of arthritis was the most frequently cited comorbidity.

Table 1 includes the mean, standard deviation, median, and interquartile range of participant demographic characteristics, physical activity, weight, and scores on PROMIS-29 domains at baseline and 18 months. Our sample reported similar median T-scores to the general USA population, with the exception of physical function at each time point (56.90 vs 50.00) and depression at 18 months (45.00 vs 50.00). Weight loss was 4.06 kg (4.45% of initial bodyweight), and ranged from 36.92 kg lost to 10.89 kg gained.

**Table 1:** Descriptive statistics of demographic characteristics, physical activity, PROMIS-29 v.1 variables, and weight at baseline and 18 months.

	Baseline (*n* = 301)			18 months (*n* = 216)		
	Mean (SD)	Median	Range^a^	Mean (SD)	Median	Range^a^
Age (years)	53.94 (6.88)	54.00	49–59	-	-	-
Number of Comorbidities	1.56 (1.31)	1.00	1–2	-	-	-
Physical Activity^b^	35.89 (19.80)	31.50	21.45–46.38	34.82 (18.36)	29.94	21.98–45.93
PROMIS-29 v.1 Domains^c^						
Anxiety	48.71 (7.08)	48.00	40.30–53.70	49.34 (7.37)	51.20	40.30–53.70
Depression	47.45 (6.80)	49.00	41.00–53.90	47.39 (6.94)	49.00	41.00–51.80
Physical Function	51.55 (6.28)	56.90	45.30–56.90	51.66 (6.86)	56.90	48.00–56.90
Pain Interference	51.71 (7.17)	53.90	41.60–55.60	52.11 (8.17)	53.90	41.60–58.50
Fatigue	51.49 (6.72)	51.00	46.65–57.00	50.33 (8.60)	51.00	46.00–57.00
Satisfaction Social Role	51.68 (6.59)	51.60	46.40–55.60	52.71 (7.11)	51.60	48.10–57.48
Sleep Disturbance	48.53 (7.33)	48.40	44.40–52.40	48.79 (7.42)	48.40	43.80–54.30
Weight (kg)	92.71 (12.90)	89.77	84.06–100.47	88.65 (14.43)	87.45	78.61–97.82

^a^Reflects the interquartile range.

^b^Measured as average daily minutes of moderate or greater intensity activity over one week.

^c^PROMIS-29 scores are T-scores with a population mean of 50.

We first analyzed the PROMIS-29 scores of women who completed the intervention phase at 18 months compared to women who did not remain in the study. At 18 months, 71 women had dropped out of the initial sample of 301 (23.6%). Women who dropped out of the study reported higher T-scores on anxiety, depression, pain interference, fatigue, and sleep disturbance, whereas they reported lower T-scores on physical function and satisfaction with social role. Results of these comparisons are included in Table 2.

**Table 2:** Comparison of baseline PROMIS-29 v 1. scores for women who completed the study compared to women who dropped out.

	Completers (*n* = 230)		Dropouts (*n* = 71)			
PROMIS-29 v.1 domains	*M*	*SD*	*M*	*SD*	*t*	*p*
Anxiety	48.83	7.03	51.68	6.72	-3.01	.003
Depression	47.50	6.77	50.36	7.74	-3.00	.003
Physical Function	51.65	6.28	49.14	6.58	2.92	.004
Pain Interference	51.86	7.25	53.88	7.31	-2.02	.044
Fatigue	51.69	6.89	54.87	6.85	-3.41	.001
Satisfaction Social Role	51.56	6.64	49.02	6.34	2.85	.005
Sleep Disturbance	48.64	7.30	50.04	6.31	2.92	.004

Complete results of all linear regression analyses are depicted in Table 3. After adjusting for age, number of comorbidities, change in physical activity from baseline, intervention group, and baseline PROMIS-29 scores, change in weight was associated with improved HRQoL in depression (*b* = 0.13, 95% CI: 0.03, 0.24, Δ*R*^2^ from model that included only adjustments = 0.02), physical function (*b* = -0.18, 95% CI: -0.28, -0.08, Δ*R*^2^ = 0.04), pain interference (*b* = 0.24, 95% CI: 0.12, 0.38, Δ*R*^2^ = 0.05), fatigue (*b* = 0.24, 95% CI: 0.12, 0.38, Δ*R*^2^ = 0.06), and satisfaction with social role (*b* = -0.22, 95% CI: -0.33, -0.11, Δ*R*^2^ = 0.05). For each significant relationship, findings were in the hypothesized direction in that weight loss was associated with improved scores on the PROMIS-29 domains such as lower depression and greater physical function.

**Table 3:** Association between percent change in weight and change in PROMIS-29 v 1.0 over 18 months.

	Coefficient			Model Fit	
PROMIS-29 v.1 domains	*b*	95% CI	P-value	*R*2 (Δ*R*^2^)	*F* (Δ*F*)
Anxiety	0.10	-0.02; 0.23	0.11	0.28 (<0.01)	12.89*** (2.62)
Depression	0.13	0.03; 0.24	0.01	0.30 (0.02)	14.09*** (6.12*)
Physical Function	-0.18	-0.28; -0.08	0.001	0.22 (0.04)	9.88*** (11.88**)
Pain Interference	0.24	0.12; 0.38	<0.001	0.24 (0.05)	9.29*** (14.52***)
Fatigue	0.24	0.12; 0.38	<0.001	0.20 (0.06)	7.56*** (14.40***)
Satisfaction Social Role	-0.22	-0.33; -0.11	<0.001	0.28 (0.05)	13.03*** (14.75***)
Sleep Disturbance	0.03	-0.07; 0.13	0.67	0.16 (<0.01)	6.86*** (0.32)

Models adjusted for age, number of comorbidities, change in physical activity from baseline, intervention group, and baseline PROMIS-29 scores. Δ*R*^2^ and Δ*F* refer to the comparison of the models that included only adjustments and then to the model that also included change in weight. Analyses include 216 women, aside from the analysis of depression which included 215 women.

*p < .05.

**p < .01.

***p < .001.

Results of the logistic regression analyses are shown in Table 4. We found a proportion of women within each of the three weight loss categories who met an MCID for each of the seven PROMIS-29 domains at 18 months. At 18 months, the significant differences in likelihood of attaining an MCID in a given PROMIS-29 domain occurred between women with < 5% weight loss and women with ≥ 10% weight loss. Women with ≥ 10% weight loss at 18 months were more likely to report a substantial improvement of at least 6 points in depression (OR = 2.47, p = .009, 95% CI: 1.24–4.94), physical function (OR = 2.83, p = .005, 95% CI: 1.43–5.59), and pain interference (OR = 2.08, p = .018, 95% CI: 1.13–3.61).

**Table 4:** Association between categorical change in weight and change in PROMIS-29 v 1. over 18 months.

	Baseline-18 months (n = 216)		
≥ 6 unit change in	Odds Ratio	95% CI	*n* (%)^a^
**Anxiety**			
Overall model χ^2^ (8) = 28.72, *p* < .001; Step with Weight Δ χ^2^ (2) = 0.26, *p* = .88			
< 5% weight loss	1		26/144 (18%)
5–9.9% weight loss	1.15	0.61–2.18	9/38 (24%)
≥ 10% weight loss	0.97	0.50–1.87	8/43 (19%)
**Depression**			
Overall model χ^2^ (8) = 53.58, *p* < .001; Step with Weight Δ χ^2^ (2) = 8.08, *p* = .02			
< 5% weight loss	1		20/143 (14%)
5–9.9% weight loss	0.71	0.34–1.50	6/38 (15%)
≥ 10% weight loss	2.47**	1.24–4.94	14/43 (33%)
**Physical Function**			
Overall model χ^2^ (8) = 61.77, *p* < .001; Step with Weight Δ χ^2^ (2) = 12.07, *p* = .002			
< 5% weight loss	1		17/144 (12%)
5–9.9% weight loss	0.77	0.38–1.59	7/38 (18%)
≥ 10% weight loss	2.83**	1.43–5.59	16/43 (37%)
**Pain Interference**			
Overall model χ^2^ (8) = 16.77, *p* = .03; Step with Weight Δ χ^2^ (2) = 8.15, *p* = .03			
< 5% weight loss	1		20/124 (14%)
5–9.9% weight loss	0.91	0.49–1.67	8/38 (21%)
≥ 10% weight loss	2.02*	1.13–3.61	14/43 (33%)
**Fatigue**			
Overall model χ^2^ (8) = 15.33, *p* = .05; Step with Weight Δ χ^2^ (2) = 1.80, *p* = .41			
< 5% weight loss	1		29/144 (20%)
5–9.9% weight loss	1.07	0.60–1.91	10/38 (26%)
≥ 10% weight loss	1.26	0.72–2.21	14/43 (33)
**Satisfaction Social Role**			
Overall model χ^2^ (8) = 11.34, *p* = .18; Step with Weight Δ χ^2^ (2) = 0.48, *p* = .79			
< 5% weight loss	1		20/144 (14%)
5–9.9% weight loss	1.12	0.55–2.92	6/38 (16%)
≥ 10% weight loss	0.77	0.36–1.68	5/43 (12%)
**Sleep Disturbance**			
Overall model χ^2^ (8) = 25.42, *p* = .001; Step with Weight Δ χ^2^ (2) = 2.41, *p* = .30			
< 5% weight loss	1		14/144 (10%)
5–9.9% weight loss	1.29	0.65–2.56	7/38 (18%)
≥ 10% weight loss	1.24	0.61–2.50	8/43 (19%)

Women were excluded from analyses if they did not complete all items for a given domain. Referent category for all analyses was the < 5% weight loss group. Analyses controlled for age, number of comorbidities, change in physical activity from baseline, intervention group, and baseline PROMIS-29 scores.

^a^Denotes the number and percentage of participants in each group who achieved a 6-unit change or greater in the desired direction.

*p < .05.

**p < .01.

## Discussion

This analysis is one of a limited number of studies that examine the association between HRQoL and weight change in healthy midlife and older women who complete a lifestyle modification intervention for weight loss and weight maintenance. Among our rural women, a loss in weight from baseline appeared to relate to improvement in five of seven PROMIS-29 v 1.0 domains, including self-reported perceptions of depression, fatigue, pain interference, physical function, and satisfaction with social roles. When examined by weight loss category, the women who attained ≥ 10% weight loss were more likely to report lower depression, higher physical function and less pain interference, than the referent group of women < 5% weight loss or weight gain.

Our retention rate of women at 18 months was 76.4% (n = 230) is relatively high, especially for purely web-based interventions [38,39]. Drop-out rates for weight loss interventions vary considerably, from 10% to 80%, depending on the duration and setting of the program [40]. As the literature suggest that factors such as poorer mental health and lower self-efficacy and activity levels are predictors of drop-outs in weight loss studies [40], it is not surprising that the baseline PROMIS-29 scores of women who dropped from the study had less desirable scores in all PROMIS-29 domains. Our findings were based on 216 women with complete data needed for the planned analyses, and as completers, these women may have been more likely to achieve weight loss success than non-completers [40].

Our findings of weight loss being associated with multiple HRQoL domains differs from outcomes of systematic reviews which indicate a higher likelihood of improvement in HRQoL physical components, with minimal or no improvements in the social or mental health components [6,11,16]. Potential reasons for differing results might be attributed to the systematic reviews that are based on a large number of studies of HRQoL following bariatric surgery, with fewer examining HRQoL after lifestyle modification approaches to weight loss as used in our study.

Our findings of improvement in multiple HRQoL domains with modest weight loss is similar to results of two lifestyle intervention studies of healthy overweight and obese women of similar age, 50–69, to our cohort [39, 40]. Ross and colleagues [41] reported an average baseline to six-month weight loss of 9.4% in rural women who completed an intensive intervention of low-calorie eating combined with 30 minutes of brisk walking. Weight loss following this intensive lifestyle approach contributed to HRQoL improvements in seven of nine components of the women’s SF-36 scores, with improvements observed in physical functioning, social functioning, and mental health. In a large randomized controlled trial, Imayama and collaborators [42] found women assigned to 12 months of a combined diet and exercise program improved in more aspects of the SF-36, including both physical and mental components, and with larger increments of improvement, compared with diet or exercise alone. In contrast, van Germert [43] found a modest weight loss of 6–7% in women ages 50–69 after a 16-week lifestyle intervention did not result in change in SF-36 components, with one exception of a positive effect in the domain of perceived health change from the prior year.

Although all three of these lifestyle studies examined outcome data from women of similar age as our study, making comparisons with our study outcomes is difficult due to differing HRQoL measures and study duration. Whereas those studies included provider-interaction as part of the interventions, our study was purely delivered by the web. Our findings of HRQoL improvements in five PROMIS-29 domains using linear regression analyses seems notable as purely web-based weight loss interventions generally result in smaller weight losses and lower levels of weight maintenance than face-to-face interventions, and our women attained a modest baseline to 18-month weight reduction of 4.06 kg (4.45%) [38]. Our logistic regression outcomes suggest that ≥ 10% body weight loss might result in meaningful change for lower depression, higher physical function and less pain interference. Two of the three lifestyle intervention studies for weight loss in midlife and older women found improved HRQoL in mental and physical components of the SF-36 [41,42], and similarly, we found improvement in depression, physical function, and pain interference using the PROMIS-29 in both linear and logistic models.

While change in weight is more likely to result in change in physical versus mental components of HRQoL, we observed a reduction in the PROMIS-29 domain of depression in women who attained greater weight loss over 18 months. PROMIS-29 scores do not signify whether women have a diagnosis of clinical depression; however, findings of women’s self-reported perceptions of depression following weight loss in our women may potentially be relevant, as obese women are particularly vulnerable to depression [44]. We observed no change in PROMIS-29 domains of anxiety and sleep disturbance, and the reasons for these findings remain unclear. Though women self-reported physician diagnoses of anxiety, depression and sleep disturbance, these were collected in one category of comorbidities (general issues), and we did not have access to women’s medical records to confirm clinical diagnoses of anxiety or sleep-related disorders, such as sleep apnea, which if present, may have influenced the women’s perceptions of these domains.

The strength of this study is it investigates HRQoL change following a lifestyle-modification weight-loss approach, using an innovative web-based delivery method to reach an understudied population of midlife and older rural women at high risk for cardiovascular disease. The study benefits from a relatively high retention rate for a weight loss and weight maintenance intervention conducted over 18 months. Objective measures of women’s weight and height as assessed by trained research nurses adds to the credibility of the weight change results. The study regression analyses conducted adjusted for many potential confounding variables such as age, number of comorbidities, change in physical activity from baseline, intervention group, and baseline PROMIS-29 scores that could alter interpretation of the findings. In addition, the study uses the relatively new PROMIS-29 HRQoL measure, which has high precision and allows for comparability across populations [21].

Similar to the reported limitations of studies that investigate the relationship between weight loss and HRQoL change [14], our sample included primarily highly educated white US women from rural communities, and therefore the generalizability of the results is uncertain. The age (40 to 69 years) of the women corresponds with menopause, a life transition that potentially influences weight loss; however, we did adjust for age in our analyses as the onset of menopause increases with age. The study inclusion and exclusion criteria, while allowing for a broad array of participation, did eliminate individuals who had specific obesity-related comorbidities, such as diabetes that required insulin.

While improvements were observed in selected PROMIS-29 domains among completers of an 18-month intervention, the clinical and public health implications of these improvements remain unclear. Women with ≥ 10% weight loss were more likely to attain a 6 point change in depression, physical function and pain interference in the desired direction, giving some confidence the weight loss resulted in meaningful change in these HRQoL domains. Our results should be interpreted with some caution as the methods for interpreting meaningful change in scores on Patient-Reported Outcomes have evolved over time, and there remains some varying opinions about interpreting HRQoL since the time when the parent study was conducted (2011–2014) [45].

The PROMIS-29 is considered a generic HRQoL instrument, which might be less sensitive to weight loss [6,12]. HRQoL instruments are grouped into two categories, generic which references an individuals’ general health state regardless of disease status (eg. SF-36, PROMIS-29), or specific which references HRQoL components most affected by a given condition (eg. MENQOL, IWQOL-Lite) [6]. Assessment of HRQoL in future weight loss studies may benefit from using both generic and obesity-specific measures as recommended by systematic reviews [6,11] and the 2014 Guidelines for Inclusion of Patient-Reported Outcomes in Clinical Trial Protocols [46]. Our parent trial from which this analysis was conducted occurred prior to the publication of these recommendations.

In conclusion, among a population of midlife and older rural women with PROMIS-29 data who completed an 18-month web-based lifestyle modification intervention for weight loss and weight maintenance, a loss in weight from baseline appeared to be associated with overall improvement in each PROMIS-29 v 1.0 domain aside from anxiety and sleep disturbance. A loss in weight of ≥ 10% from baseline increased the likelihood of obtaining improvements with reduced depression and pain interference and increased physical function. Findings that weight loss is associated with improved HRQoL may positively influence a woman’s adherence to lifestyle modification weight loss and weight maintenance programs.

## Supporting information

S1 Data This is the data file. (SAV)
